# Prognostic Implication of Non-Obstructive Coronary Lesions: A New Classification in Different Settings

**DOI:** 10.3390/jcm10091863

**Published:** 2021-04-25

**Authors:** Jorge Rodríguez-Capitán, Andrés Sánchez-Pérez, Sara Ballesteros-Pradas, Mercedes Millán-Gómez, Rosa Cardenal-Piris, Manuel Oneto-Fernández, Lola Gutiérrez-Alonso, Ricardo Rivera-López, Agustín Guisado-Rasco, Macarena Cano-García, Mario Gutiérrez-Bedmar, Manuel Jiménez-Navarro

**Affiliations:** 1 Área del Corazón, UMA Campus de Teatinos S/N, Hospital Universitario Virgen de la Victoria, CIBERCV, IBIMA, 29010 Málaga, Spain; capijorge@hotmail.com (J.R.-C.); AsanperHVS@outlook.com (A.S.-P.); mercedesmillang@gmail.com (M.M.-G.); 2 Hospital Universitario Virgen de Valme, Carretera de Cádiz Km. 548.9, 41014 Sevilla, Spain; sara_mbp@hotmail.com; 3 Hospital Universitario Juan Ramón Jiménez, Ronda Norte S/N, 21005 Huelva, Spain; rcpiris@gmail.com; 4 Hospital Universitario Reina Sofía, Avenida Menéndez Pidal, 14004 Córdoba, Spain; mjoneto@outlook.com; 5 Hospital Universitario Puerta del Mar, Cádiz, Avenida Ana de Viya 21, 11009 Cádiz, Spain; lolagutialonso@gmail.com; 6 Servicio de Cardiología, Instituto de Investigación Biosanitaria (Ibs), Hospital Universitario Virgen de las Nieves, Avenida de las Fuerzas Armadas 2, 18014 Granada, Spain; rfriveralopez@gmail.com; 7 Hospital Universitario Virgen del Rocío, CIBERCV, Avenida Manuel Siurot S/N, 41013 Sevilla, Spain; aguiras@hotmail.com; 8 Hospital Regional Universitario de Málaga, Avenida de Carlos Haya 84, 29010 Málaga, Spain; macarenacanogarcia@hotmail.com; 9 Department of Preventive Medicine and Public Health, School of Medicine, Campus de Teatinos S/N, University of Málaga, 29010 Málaga, Spain

**Keywords:** coronary angiography, coronary artery disease, sex, diabetes mellitus, acute coronary syndrome

## Abstract

The clinical significance of non-obstructive coronary artery disease is the subject of debate. Our objective was to evaluate the long-term cardiovascular prognosis associated with non-obstructive coronary artery disease in patients undergoing coronary angiography, and to conduct a stratification by sex, diabetes, and clinical indication. We designed a multi-centre retrospective longitudinal observational study of 3265 patients that were classified into three groups: normal coronary arteries (lesion <20%, 1426 patients), non-obstructive coronary artery disease (20–50%, 643 patients), and obstructive coronary artery disease (>70%, 1196 patients). During a mean follow-up of 43 months, we evaluated a combined cardiovascular event: acute myocardial infarction, stroke, hospitalization for heart failure, or cardiovascular death. Multivariable-adjusted Cox proportional hazard models showed a worse prognosis in patients with non-obstructive coronary artery disease, in comparison with patients of normal coronary arteries group, in the total population (hazard ratio 1.72, 95% confidence interval 1.23–2.39; p for trend <0.001), in non-diabetics (hazard ratio 2.12, 95% confidence interval: 1.40–3.22), in women (hazard ratio 1.75, 95% confidence interval 1.10–2.77), and after acute coronary syndrome (hazard ratio 2.07, 95% confidence interval 1.25–3.44). In conclusion, non-obstructive coronary artery disease is associated with an impaired long-term cardiovascular prognosis. This association held for non-diabetics, women, and after acute coronary syndrome.

## 1. Introduction

The absence of obstructive coronary artery disease (CAD) is a frequent finding in patients undergoing angiography both in the context of chronic coronary syndrome [1,2] and acute coronary syndrome (ACS) [3,4].

Although it was initially proposed that the absence of obstructive CAD could constitute an unequivocal sign of a good cardiovascular prognosis [5], subsequent evidence has increasingly suggested that the presence of non-obstructive CAD confers an adverse prognosis when compared to the prognosis in the absence of CAD [1,6,7,8]. In any case, the prognostic value of non-obstructive CAD remains unclear for various reasons, particularly in the mid- and long-term. Firstly, the published studies are heterogeneous in their definition of the absence of CAD, and in the diagnostic method employed (computed tomography or invasive angiography) [2]. Secondly, the majority of studies focus on a specific clinical profile of patient. Thus, the evidence for an adverse prognosis has essentially focused on the context of chronic coronary syndrome [1,2,6,7,8], with very little data available regarding ACS [4,9]. Likewise, data regarding the prognostic value of non-obstructive CAD among diabetics are scarce [10], despite the fact that obstructive CAD has shown a particularly adverse prognosis among this population [11]. Thirdly, in addition to the substantial differences in ischaemic heart disease between the sexes [12], there is controversy about whether the prognostic importance of non-obstructive CAD is similar for both sexes [13].

Thus, the objective of this study was to assess the long-term (minimum two years) cardiovascular prognosis that was associated with non-obstructive CAD, as compared to the absence of CAD and to the presence of obstructive CAD, in an extensive sample of patients undergoing invasive angiography for screening of ischaemic heart disease, stratifying by sex, the presence of diabetes mellitus, and the clinical indication for the angiography.

## 2. Materials and Methods

We performed a multi-centre, retrospective longitudinal observational study, with the participation of eight hospitals from different provinces in the south of Spain. The patients that were selected as eligible were those over the age of 18 who had undergone invasive coronary angiography for suspected myocardial ischaemia due to the presence of symptoms suggestive of any of the following clinical pictures: chest pain/ chronic coronary syndrome, non-ST segment elevation myocardial infarction, or ST segment elevation myocardial infarction. Between January 2011 and December 2015, each centre was asked to include all patients with the stated characteristics who underwent a coronary angiography that found an absence of CAD greater than 50%. For each patient included with these characteristics, we asked the centres to include the next patient whose angiography showed an obstructive lesion that was greater than 70%. The angiographies were evaluated at each centre by the interventional cardiologist who performed the procedure, and the extent of CAD was determined by visual estimation.

The exclusion criteria were any of the following: prior percutaneous or surgical revascularisation, indication for angiography other than suspected myocardial ischaemia, significant (>50%) disease of the left main coronary artery, and the presence of CAD classified as between 50% and 70% in any vessel except the left main coronary artery in the absence of other CAD >70%.

The study was performed in accordance with the Declaration of Helsinki and it was approved by the ethic committee at Hospital Universitario Virgen de la Victoria. The need for individual patient consent was waived.

According to the angiography findings, patients were classified into three groups: normal coronary arteries (the absence of lesion or less than 20% lesion), non-obstructive CAD (lesion between 20% and 50%), and obstructive CAD (lesion higher than 70%). We pretended to classify, according to the mentioned protocol, as obstructive the lesions for which the previous bibliography showed consensus that they should be taken as obstructive (>70%) and classify as non-obstructive those lesions that are clearly taken as non-obstructive in the previous studies (20–50%). Those patients that had a maximum lesion between 50 to 70% were excluded; they are classified as obstructive in some studies [1] and non-obstructive in others [7,10].

In order to assess the validity of the measurements, 300 angiographies were selected at random and sent to the study’s coordinating centre, where they were jointly assessed by two expert cardiologists (AS and MJN), who evaluated the extent and severity of the CAD. The two cardiologists were blind to previous measurements made by the participating centres. From the medical records, we collected data about demographic variables, cardiovascular risk factors (smoker, diabetes mellitus, high blood pressure, dyslipidemia), history of heart failure, kidney failure, atrial fibrillation, and left ventricular function. We took the definition for diabetes mellitus as a fasting plasma blood glucose level ≥126 mg/dL after at least 8 h fasting, and/or a presence of glycated haemoglobin greater than 6.5%, and/or the presence of a prior diagnosis of diabetes mellitus and/or treatment with anti-diabetic medication. High blood pressure was defined as a prior diagnosis of arterial hypertension and/or the use of antihypertensives and/or blood pressure ≥140/90 mmHg; dyslipidemia as a total cholesterol >200 mg/dL or the use of lipid-lowering agent therapy; and, kidney failure as glomerular filtration <60 mL/min.

The primary endpoint was a composite of cardiovascular events, being defined as acute myocardial infarction, stroke, hospitalization for heart failure, or death from cardiovascular cause. Acute myocardial infarction was defined as a troponin elevation ≥2 times the upper limit of normal, with or without the electrocardiographic changes typical of ischaemia in the context of clinical symptoms compatible with ACS. Death was considered to be cardiovascular in origin unless an unequivocal noncardiac cause was observed. A longitudinal follow-up was conducted, and it was considered to be complete if it lasted at least 24 months or until the onset of an event. Clinical events during follow-up were assessed for the whole group as well as by strata according to the presence or not of diabetes mellitus, by sex, and by the clinical indication for the angiography (chest pain/ chronic coronary syndrome or unstable patients with myocardial infarction with or without ST segment elevation).

Baseline clinical and demographic characteristics were summarized by means and standard deviations for continuous variables or count (%) for categorical variables. Adjusted levels of covariates across groups according to the degree of CAD were estimated using analysis of variance. Polynomial contrast (linear trend) was used to evaluate the association of the adjusted levels with degree of CAD. Cohen’s Kappa was used to measure the agreement of measures of degree of CAD. We estimated the risk of event according to the percentage of CAD using multivariate Cox regression models, taking the normal coronary arteries group as the reference. To do this, two models were created, one adjusted by age and sex, and another additionally adjusted by smoking habit, dyslipidemia, diabetes, heart failure, kidney failure, indication for angiography (chest pain/ chronic coronary syndrome as opposed to ACS), atrial fibrillation, and left ventricular ejection fraction. Following the multivariate Cox proportional hazard model, the adjusted curves were plotted by a degree of CAD. Pre-specified subgroup analyses were conducted according to diabetes, sex, and indication for angiography. All of the statistical tests were two-sided, and p-values of <0.05 were considered to be statistically significant. All statistical analyses were conducted using Stata 15.1 (Stata Corp).

## 3. Results

### 3.1. General Characteristics

We included a total 3265 patients who had undergone angiography in one of the participating centres between January 2011 and December 2015. Of these patients, 1426 (43.7%) belonged to the normal coronary arteries group, 643 (19.7%) to the non-obstructive CAD group, and 1196 (36.6%) to the obstructive CAD group.

Of the total number of patients in our sample, 44.1% were women and 31.1% presented diabetes mellitus. The clinical symptoms that led to the indication for the angiography were a study of chest pain/ chronic coronary syndrome in 52.7% of patients, whereas, for 47.3%, it was ACS (35% non-ST segment elevation myocardial infarction and 12.3% ST segment elevation myocardial infarction). Table 1 shows the baseline characteristics that were adjusted by age, sex, and the patient’s hospital centre according to the degree of the coronary artery involvement. The severity of the CAD was found to be directly associated with age, smoking habit, and the presence of diabetes, kidney failure, or ACS. On the other hand, the female sex, the presence of atrial fibrillation, or a normal left ventricular ejection fraction were inversely associated with the severity of CAD. There was high concordance between the participating centres and the two expert cardiologists: Kappa = 0.827 (95% CI: 0.768–0.882).

### 3.2. Follow-Up of the Whole Sample and the Subgroups

Table 2 shows the events, incidence rates, and rate ratio (RR), according to the severity of the lesion for the whole sample and by subgroups (diabetes, sex and indication for angiography) during the study period. Follow-up to two years, or until the combined event if one occurred before the end of that period, was completed for 97.4% of the sample (3179 patients). The median follow-up was 43 months (interquartile range 33–56 months). During this period, there were 412 (12.6%) events, of which 86 (86/1426; 6%) were in the normal coronary arteries group, 78 (78/643; 12.1%) in non-obstructive CAD group, and 248 (248/1196; 20.7%) in the obstructive CAD group.

We observed a clear direct relationship between the degree of CAD and the incidence rate of events in the total population, and found a progressive increase in the risk of events as the severity of CAD increased (RR = 1.99, 95% CI: 1.45–2.74 and RR = 3.74, 95% CI: 2.91–4.83 for non-obstructive CAD and obstructive CAD, respectively, vs. normal coronary arteries). This direct relationship between the incidence of events and the severity of CAD held in the stratification by diabetes, sex, and indication for angiography, with the highest risk of events being found for the non-obstructive CAD group among non-diabetics and women (RR = 2.38, 95% CI:1.59–3.57, and RR = 2.22, 95% CI: 1.41–3.48, respectively) (Table 2).

#### Severity of the CAD and Risk of Cardiovascular Event

**Figure 1:**
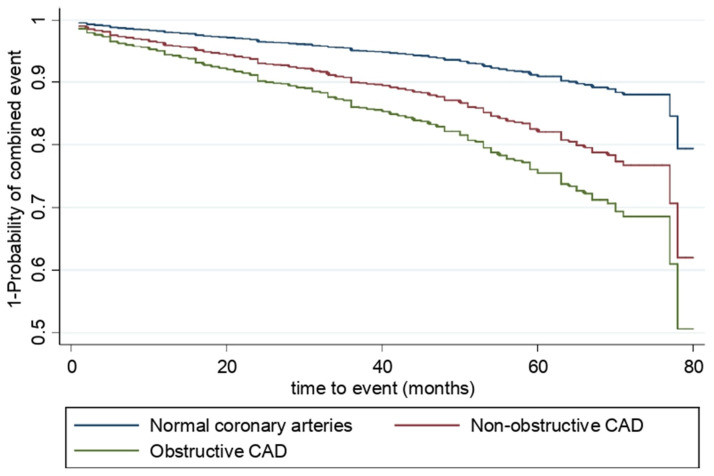
Adjusted Kaplan-Meier curves for combined event (acute myocardial infarction, stroke, hospitalization for heart failure or cardiovascular death) according to the degree of CAD using Cox proportional hazards regression. The models are adjusted by smoker, high blood pressure, dyslipidemia, diabetes, heart failure, kidney failure, indication for angiography, atrial fibrillation, and left ventricular ejection fraction. CAD: coronary artery disease.

**Figure 2:**
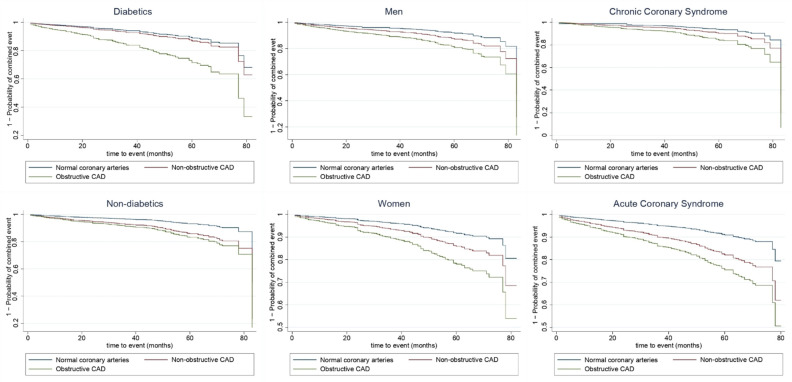
Adjusted Kaplan–Meier curves for combined event (acute myocardial infarction, stroke, hospitalization for heart failure or cardiovascular death) according to the degree of CAD using Cox proportional hazards regression stratified by diabetes, sex and indication for angiography. Models are adjusted by smoker, high blood pressure, dyslipidemia, diabetes (except for models stratified by diabetes), heart failure, kidney failure, indication for angiography (except for models stratified by indication for angiogrphy), atrial fibrillation, and left ventricular ejection fraction. CAD: coronary artery disease.

Table 3, Figure 1, and Figure 2 present the associations of degree of CAD with the risk of cardiovascular event. In the total population, the adjusted Cox proportional hazard models showed that the non-obstructive CAD and obstructive CAD participants presented a significantly higher risk of developing cardiovascular events than those with normal coronary arteries (Hazard Ratio (HR) = 1.72, 95% CI: 1.23–2.39 and HR = 2.71, 95% CI: 2.03–3.62 respectively; *p* for trend <0.001). In the stratified analysis for diabetes, sex, and indication for angiography (Table 3 and Figure 2), a direct relationship could still be observed between the severity of CAD and the risk of a cardiovascular event (*p* for trend <0.001 in all of the models). However, in comparing the non-obstructive CAD group of patients to patients with normal coronary arteries, a higher risk of cardiovascular event was only found for non-diabetics (HR = 2.12, 95% CI: 1.40–3.22), women (HR = 1.75, 95% CI:1.10–2.77), and patients with ACS (HR = 2.07, 95%: 1.25–3.44). 

## 4. Discussion

In this study, we found that, after a long-term follow-up of a large sample of patients who underwent coronary angiography for suspected ischaemic heart disease, the presence of non-obstructive CAD (lesion 20–50%), as compared with the finding of normal coronary arteries (absence of CAD or lesion <20%), was associated with a higher risk of cardiovascular events in the whole study population. In the analysis by subgroups, this association also held for non-diabetics, women, and patients with ACS as an indication for angiography.

Regarding the clinical profile of patients, the singularity of this study consists in its broad representation of both stable and unstable patients, unlike the majority of previous studies that have focused on a specific clinical profile, frequently chronic coronary syndrome [1,2,4,6,7,8,9]. With reference to the exposure evaluated, the particularity of this study consists in its classification of cases into three degrees of coronary artery involvement: obstruction up to 20%, from 20% to 50%, or greater than 70%, and its exclusion of borderline cases (lesion 50–70%). In these borderline cases, for which it is particularly difficult to assess the clinical implications, the intracoronary diagnostic techniques that are currently little employed in clinical practice could be of use [14].

The analysis of events by indication for the angiography showed a direct association between the degree of CAD and development of a combined event in unstable patients. There have been numerous series that show an association between non-obstructive CAD and an adverse cardiovascular prognosis in stable patients [1,7,8,15,16]. However, there is very little published evidence on the prognosis of non-obstructive CAD in ACS, as a recent meta-analysis showed [9]. With reference to previous evidence about ACS, our study contributes two differentiating characteristics: first, it shows the results for patients in daily clinical practice, as opposed to other studies that show selected patients from clinical trials [4]. Secondly, it is based on a novel classification of CAD into three groups, excluding patients with borderline CAD (50–70%) who are more difficult to correctly classify. Evidence for the prognostic value of non-obstructive CAD in ACS follows the line of authors who propose a new paradigm in the pathophysiology and natural history of ACS, in which global atherosclerotic burden, rather than vulnerable plaque, acquires particular importance [17,18].

This study shows that women have a higher percentage of cardiovascular events as the degree of coronary stenosis increases. The significance of non-obstructive CAD depending on sex is a highly topical subject [8], and it has been suggested that microvascular dysfunction is a pathophysiological mechanism that is particularly relevant in the female sex. There is a consensus that the absence of significant CAD is more frequent among women, but, to date, controversy continues regarding whether the prognostic value of non-obstructive CAD is different for women and men. This is due, in great part, to the under-representation of women in many of the studies and the lack of studies specifically directed at this sex [8,15,19,20]. For these reasons, the need exists to undertake studies that aimed at the specific assessment of each sex [21], as we have done in the present study. Thus, in our series, women present a higher risk of events, which increases with the degree of coronary stenosis, which would seem to support the atherosclerotic theory in the same way it does for men.

In the stratification according to presence of diabetes, the adjusted model for risk factors and comorbidities shows that the presence of non-obstructive CAD is associated with an increase of events in follow-up as compared to normal coronary arteries in non-diabetic patients. There are relatively few studies on the prognostic value of non-obstructive CAD in diabetics. To date, the published findings have shown that the extent of atherosclerosis predicts the development of cardiovascular events in asymptomatic diabetics [10] and in diabetics with suspected CAD [22]. However, to our knowledge, no solid evidence has been published to date showing an adverse prognosis associated to the presence of non-obstructive CAD in diabetics. One possible explanation for this is that diabetics present a high cardiovascular risk, even in cases with an absence of CAD, as, in fact, occurs in our research with an incidence rate of 24.1 events per 1000 people-year, similar to the cardiovascular risk that non-obstructive CAD confers. Another possible explanation is the pathophysiology of diabetes itself, which may trigger vascular damage due to mechanisms that are different to those of non-diabetic patients [23].

There are some limitations to our study. This is a retrospective study; it did not collect data relating to patient treatment, which could have had an influence on clinical prognosis. We selected a sample of symptomatic patients with suspected ischaemic heart disease in a region that has a lower prevalence of ischaemic heart disease and a lower number of angiographies per million inhabitants than other European countries. In addition, our sample may have included patients who presented both appropriate and inappropriate referrals. In this retrospective study, only visual estimation determined the presence and extent of CAD, as it was usually performed in the participating centres during the inclusion period. The angiographies were assessed at each centre by the interventional cardiologist who performed the procedure, which conditions an inter-observer variability in quantifying the CAD, although the concordance between the participating centres and the two expert cardiologists was high. The retrospective nature of the study also limited the classification of coronary arteries with non-obstructive disease, which was necessary to perform according to the habitual practice of each study centre, distinguishing between normal coronary arteries (without CAD, or lesion <20%) or non-obstructive CAD (lesion 20–50%) without the imposition of further cut-off points in the 0 to 50% spectrum. The number of vessels involved was not classified, nor was total number of lesions observed in the angiography, unlike some other studies [6,8]. Those patients that had lesions between 50 to 70% (and no lesions bigger than 70%) were excluded from the study, which limits our findings. However, instead, those patients with non-obstructive and those with obstructive lesions were accurately defined. Although the total number of patients included was large (3265 patients), only 643 belonged to non-obstructive CAD group, and the relatively small size of this group could limit the results obtained.

## 5. Conclusions

In conclusion, this study shows that the presence of non-obstructive CAD (lesion 20–50%) is associated with a worse long-term cardiovascular prognosis as compared to the presence of normal coronary arteries (the absence of CAD or lesion <20%), in a wide sample of patients of both sexes undergoing coronary angiography in both stable and unstable situations. This adverse cardiovascular prognosis held after adjustment for the main long-term prognostic factors. In the analysis by subgroups, this association also held for non-diabetics, women, and patients ACS as indication for angiography. The presents findings have potentially significant clinical implications in the management of patients with non-obstructive CAD.

## Figures and Tables

**Table 1:** Adjusted baseline characteristics of the participants, according to the degree of the coronary artery involvement.

Characteristic	<20% (N = 1426)	20–50% (N = 643)	>70% (N = 1196)	*p*-Trend *
Age ^†^ (years)	61.3	65.9	65.4	<0.001
Sex ^‡^ (women)	59.5	43.2	26.2	<0.001
Smoker	32.7	39.8	44.5	<0.001
High blood pressure	65.8	69.9	64.1	0.352
Diabetes mellitus	28.0	29.2	35.7	<0.001
Dyslipidemia	47.5	51.8	50.0	0.233
Heart failure	4.5	3.1	5.3	0.337
Kidney failure	5.3	7.6	12.4	<0.001
Acute coronary syndrome	34.6	38.4	67.2	<0.001
Atrial fibrillation	13.5	10.3	6.0	<0.001
Left ventricular ejection fraction				
Normal	90.2	89.3	71.8	<0.001
Mild dysfunction	5.7	5.8	16.0	<0.001
Severe dysfunction	2.0	2.8	10.0	<0.001

Baseline characteristics are adjusted by age, sex and the patient’s hospital centre. Data are given as %, or as mean for age. * *p* for linear trend was tested by ANOVA. ^†^ Adjusted by sex and centre. ^‡^ Adjusted by age and centre.

**Table 2:** The risk of cardiovascular event according to the degree of coronary artery disease.

	<20% (N = 1426)	20–50% (N = 643)	>70% (N = 1196)
**TOTAL POPULATION**			
Number of person-year follow-up	5291.0	2406.6	4084.3
Number of events	86	78	248
Incidence rate per 1000 person-year	16.3 (13.2–20.1)	32.4 (26.0–40.5)	60.7 (53.6–68.8)
Rate ratio	Reference	1.99 (1.45–2.74)	3.74 (2.91–4.83)
STRATIFIED BY DIABETES			
Diabetics			
Number of person-year follow-up	1452.6	732.6	1420.8
Number of events	35	25	117
Incidence rate per 1000 person-year	24.1 (17–33.6)	34.1 (23.1–50.5)	82.3 (68.7–98.7)
Rate ratio	Reference	1.42 (0.81–2.43)	3.42 (2.33–5.14)
Non-diabetics			
Number of person-year follow-up	3838.4	1674.0	2663.5
Number of events	51	53	131
Incidence rate per 1000 person-year	13.3 (10.1–17.5)	31.7 (24.2–41.4)	49.2 (41.4–58.4)
Rate ratio	Reference	2.38 (1.59–3.57)	3.70 (2.66–5.22)
STRATIFIED BY SEX			
Men			
Number of person-year follow-up	2155.1	1341.9	2998.2
Number of events	37	41	175
Incidence rate per 1000 person-year	17.2 (12.4–23.7)	30.6 (22.5–41.5)	58.4 (50.3–67.7)
Rate ratio	Reference	1.78 (1.11–2.85)	3.40 (2.37–4.99)
Women			
Number of person-year follow-up	3135.9	1064.7	1086.1
Number of events	49	37	73
Incidence rate per 1000 person-year	15.6 (11.8–20.7)	34.8 (25.2–48.0)	67.2 (53.4–84.5)
Rate ratio	Reference	2.22 (1.41–3.48)	4.30 (2.95–6.31)
STRATIFIED BY INDICATION FOR ANGIOGRAPHY			
Chronic coronary syndrome			
Number of person-year follow-up	3582.8	1501.4	1305.7
Number of events	52	40	62
Incidence rate per 1000 person-year	14.5 (11.1–19.0)	26.6 (19.5–36.3)	47.5 (37.0–60.9)
Rate ratio	Reference	1.84 (1.18–2.83)	3.27 (2.23–4.82)
Acute coronary syndrome			
Number of person-year follow-up	1684.3	897.4	2756.7
Number of events	34	38	184
Incidence rate per 1000 person-year	20.2 (14.4–28.3)	42.3 (30.8–58.2)	66.7 (57.8–77.1)
Rate ratio	Reference	2.10 (1.29–3.43)	3.31 (2.28–4.92)

Incidence rates and rate ratios are expressed as number (95% confidence interval).

**Table 3:** Hazard ratios of cardiovascular event according to the degree of coronary artery disease.

	<20% (N = 1426)	20–50% (N = 643)	>70% (N = 1196)	*p*-Trend
TOTAL POPULATION				
Events/Number of patients	84/1426	78/643	249/1196	
Model 1 *	Reference	1.86 (1.34–2.56)	3.57 (2.75–4.62)	<0.001
Model 2 ^†^	Reference	1.72 (1.23–2.39)	2.71 (2.03–3.62)	<0.001
STRATIFIED BY DIABETES				
Diabetics				
Events/Number of patients	35/390	25/195	118/429	
Model 1 *	Reference	1.40 (0.82–2.37)	3.44 (2.31–5.14)	<0.001
Model 2 ^†^	Reference	1.21 (0.69–2.13)	2.87 (1.83–4.50)	<0.001
Non-diabetics				
Events/Number of patients	51/1036	53/448	131/767	
Model 1 *	Reference	2.18 (1.47–3.25)	3.46 (2.45–4.88)	<0.001
Model 2 ^†^	Reference	2.12 (1.40–3.22)	2.57 (1.75–3.76)	<0.001
STRATIFIED BY SEX				
Men				
Events/Number of patients	37/599	41/353	176/873	
Model 1 *	Reference	1.69 (1.08–2.67)	3.32 (2.31–4.75)	<0.001
Model 2 ^†^	Reference	1.59 (0.98–2.58)	2.47 (1.65–3.68)	<0.001
Women				
Events/Number of patients	49/827	37/290	73/323	
Model 1 *	Reference	2.00 (1.29–3.11)	3.67 (2.50–5.37)	<0.001
Model 2 ^†^	Reference	1.75 (1.10–2.77)	2.85 (1.87–4.35)	<0.001
STRATIFIED BY INDICATION FOR ANGIOGRAPHY				
Chronic coronary syndrome				
Events/Number of patients	52/930	40/390	62/393	
Model 1 *	Reference	1.65 (1.07–2.54)	2.76 (1.84–4.14)	<0.001
Model 2 ^†^	Reference	1.55 (0.98–2.44)	2.58 (1.68–3.96)	<0.001
Acute coronary syndrome				
Events/Number of patients	34/489	38/251	185/795	
Model 1 *	Reference	2.23 (1.38–3.61)	3.63 (2.41–5.46)	<0.001
Model 2 ^†^	Reference	2.07 (1.25–3.44)	2.95 (1.92–4.54)	<0.001

Model 1 and model 2 are expressed as hazard ratio (95% confidence interval). * Model 1 is adjusted by center, age, and sex (except for models stratified by sex). ^†^ Model 2 is additionally adjusted by smoker, high blood pressure, dyslipidemia, diabetes (except for models stratified by diabetes), heart failure, kidney failure, indication for angiography (except for models stratified by indication for angiography), atrial fibrillation, and left ventricular ejection fraction.

## Data Availability

The data presented in this study are available on request from the corresponding author. The data are not publicly available due to the recommendations of the ethic committee.

## References

[1] Finck T., Hardenberg J., Will A., Hendrich E., Haller B., Martinoff S., Hausleiter J., Hadamitzky M. (2019). 10-Year Follow-Up After Coronary Computed Tomography Angiography in Patients with Suspected Coronary Artery Disease. J. Am. Coll. Cardiol. Cardiovasc. Imaging.

[2] Radico F., Zimarino M., Fulgenzi F., Ricci F., Di Nicola M., Jespersen L., Chang S.M., Humphries K.H., Marzilli M., De Caterina R. (2018). Determinants of long-term clinical outcomes in patients with angina but without obstructive coronary artery disease: A systematic review and meta-analysis. Eur. Heart J..

[3] Safdar B., Spatz E.S., Dreyer R.P., Beltrame J.F., Lichtman J.H., Spertus J.A., Reynolds H.R., Geda M., Bueno H., Dziura J.D. (2018). Presentation, Clinical Profile, and Prognosis of Young Patients with Myocardial Infarction with Nonobstructive Coronary Arteries (MINOCA): Results from the VIRGO Study. J. Am. Heart Assoc..

[4] Bugiardini R., Manfrini O., De Ferrari G.M. (2006). Unanswered questions for management of acute coronary syndrome: Risk stratification of patients with minimal disease or normal findings on coronary angiography. Arch. Intern. Med..

[5] Kemp H.G., Kronmal R.A., Vlietstra R.E., Frye R.L. (1986). Seven year survival of patients with normal or near normal coronary arteriograms: A CASS registry study. J. Am. Coll. Cardiol..

[6] Maddox T.M., Stanislawski M.A., Grunwald G.K., Bradley S.M., Ho P.M., Tsai T.T., Patel M.R., Sandhu A., Valle J., Magid D.J. (2014). Nonobstructive coronary artery disease and risk of myocardial infarction. JAMA.

[7] Nakazato R., Arsanjani R., Achenbach S., Gransar H., Cheng V.Y., Dunning A., Lin F.Y., Al-Mallah M., Budoff M.J., Callister T.Q. (2014). Age-related risk of major adverse cardiac event risk and coronary artery disease extent and severity by coronary CT angiography: Results from 15,187 patients from the International Multisite CONFIRM Study. Eur. Heart J. Cardiovasc. Imaging.

[8] Schulman-Marcus J., Hartaigh B.Ó., Gransar H., Lin F., Valenti V., Cho I., Berman D., Callister T., DeLago A., Hadamitzky M. (2016). Sex-Specific Associations Between Coronary Artery Plaque Extent and Risk of Major Adverse Cardiovascular Events: The CONFIRM Long-Term Registry. J. Am. Coll. Cardiol. Cardiovasc. Imaging.

[9] Wang Z.J., Zhang L.L., Elmariah S., Han H.Y., Zhou Y.J. (2017). Prevalence and prognosis of nonobstructive coronary artery disease in patients undergoing coronary angiography or coronary computed tomography angiography: A meta-analysis. Mayo Clin. Proc..

[10] Kang S.H., Park G.M., Lee S.W., Yun S.C., Kim Y.H., Cho Y.R., Park H.W., Suh J., Yang D.H., Kang J.W. (2016). Long-Term Prognostic Value of Coronary CT Angiography in Asymptomatic Type 2 Diabetes Mellitus. J. Am. Coll. Cardiol. Cardiovasc. Imaging.

[11] Rana J.S., Dunning A., Achenbach S., Al-Mallah M., Budoff M.J., Cademartiri F., Callister T.Q., Chang H.J., Cheng V.Y., Chinnaiyan K. (2012). Differences in prevalence, extent, severity, and prognosis of coronary artery disease among patients with and without diabetes undergoing coronary computed tomography angiography: Results from 10,110 individuals from the CONFIRM (COronary CT Angiography EvaluatioN For Clinical Outcomes): An InteRnational Multicenter Registry. Diabetes Care.

[12] Mieres J.H., Bonow R.O. (2016). Ischemic Heart Disease in Women: A Need for Sex-Specific Diagnostic Algorithms. J. Am. Coll. Cardiol. Cardiovasc. Imaging.

[13] Sharaf B., Wood T., Shaw L., Johnson B.D., Kelsey S., Anderson R.D., Pepine C.J., Bairey Merz C.N. (2013). Adverse outcomes among women presenting with signs and symptoms of ischemia and no obstructive coronary artery disease: Findings from the National Heart, Lung, and Blood Institute-sponsored Women’s Ischemia Syndrome Evaluation (WISE) angiographic core laboratory. Am. Heart J..

[14] Lee J.M., Choi K.H., Koo B.K., Zhang J., Han J.K., Yang H.M., Park K.W., Song Y.B., Hahn J.Y., Choi S.H. (2020). Intravascular ultrasound or optical coherence tomography-defined anatomic severity and hemodynamic severity assessed by coronary physiologic indices. Rev. Esp. Cardiol..

[15] Ouellette M.L., Löffler A.I., Beller G.A., Workman V.K., Holland E., Bourque J.M. (2018). Clinical Characteristics, Sex Differences, and Outcomes in Patients with Normal or Near-Normal Coronary Arteries, Non-Obstructive or Obstructive Coronary Artery Disease. J. Am. Heart Assoc..

[16] Jespersen L., Abildstrom S.Z., Hvelplund A., Madsen J.K., Galatius S., Pedersen F., Hojberg S., Prescott E. (2014). Burden of hospital admission and repeat angiography in angina pectoris patients with and without coronary artery disease: A registry-based cohort study. PLoS ONE.

[17] Libby P., Pasterkamp G. (2015). Requiem for the ‘vulnerable plaque’. Eur. Heart J..

[18] Stone G.W., Maehara A., Lansky A.J., de Bruyne B., Cristea E., Mintz G.S., Mehran R., McPherson J., Farhat N., Marso S.P. (2011). PROSPECT Investigators. A prospective natural-history study of coronary atherosclerosis. N. Engl. J. Med..

[19] Sedlak T.L., Lee M., Izadnegahdar M., Merz C.N., Gao M., Humphries K.H. (2013). Sex differences in clinical outcomes in patients with stable angina and no obstructive coronary artery disease. Am. Heart J..

[20] Min J.K., Dunning A., Lin F.Y., Achenbach S., Al-Mallah M., Budoff M.J., Cademartiri F., Callister T.Q., Chang H.J., Cheng V. (2011). CONFIRM Investigators. Age- and sex-related differences in all-cause mortality risk based on coronary computed tomography angiography findings results from the International Multicenter CONFIRM (Coronary CT Angiography Evaluation for Clinical Outcomes: An International Multicenter Registry) of 23,854 patients without known coronary artery disease. J. Am. Coll Cardiol..

[21] Pepine C.J., Ferdinand K.C., Shaw L.J., Light-McGroary K.A., Shah R.U., Gulati M., Duvernoy C., Walsh M.N., Bairey Merz C.N. (2015). ACC CVD in Women Committee. Emergence of Nonobstructive Coronary Artery Disease: A Woman’s Problem and Need for Change in Definition on Angiography. J. Am. Coll. Cardiol..

[22] Andreini D., Pontone G., Mushtaq S., Bertella E., Conte E., Baggiano A., Veglia F., Agostoni P., Annoni A., Formenti A. (2013). Prognostic value of multidetector computed tomography coronary angiography in diabetes: Excellent long-term prognosis in patients with normal coronary arteries. Diabetes Care.

[23] Poznyak A., Grechko A.V., Poggio P., Myasoedova V.A., Alfieri V., Orekhov A.N. (2020). The Diabetes Mellitus-Atherosclerosis Connection: The Role of Lipid and Glucose Metabolism and Chronic Inflammation. Int. J. Mol. Sci..

